# Automated Sagittal Skeletal Classification of Children Based on Deep Learning

**DOI:** 10.3390/diagnostics13101719

**Published:** 2023-05-12

**Authors:** Lan Nan, Min Tang, Bohui Liang, Shuixue Mo, Na Kang, Shaohua Song, Xuejun Zhang, Xiaojuan Zeng

**Affiliations:** 1 College of Stomatology, Guangxi Medical University, Nanning 530021, China; yimuxiao@126.com (L.N.); tangmin622@163.com (M.T.); msx0226@163.com (S.M.); kangna78@126.com (N.K.); ssh_001@163.com (S.S.); 2 School of Computer, Electronics and Information, Guangxi University, Nanning 530004, China; lh_hui0631@163.com; 3 Guangxi Health Commission Key Laboratory of Prevention and Treatment for Oral Infectious Diseases, Nanning 530021, China; 4 Guangxi Key Laboratory of Oral and Maxillofacial Rehabilitation and Reconstruction, Nanning 530021, China

**Keywords:** deep learning, orthodontics, pediatric, convolutional neural networks, diagnostic systems introduction

## Abstract

Malocclusions are a type of cranio-maxillofacial growth and developmental deformity that occur with high incidence in children. Therefore, a simple and rapid diagnosis of malocclusions would be of great benefit to our future generation. However, the application of deep learning algorithms to the automatic detection of malocclusions in children has not been reported. Therefore, the aim of this study was to develop a deep learning-based method for automatic classification of the sagittal skeletal pattern in children and to validate its performance. This would be the first step in establishing a decision support system for early orthodontic treatment. In this study, four different state-of-the-art (SOTA) models were trained and compared by using 1613 lateral cephalograms, and the best performance model, Densenet-121, was selected was further subsequent validation. Lateral cephalograms and profile photographs were used as the input for the Densenet-121 model, respectively. The models were optimized using transfer learning and data augmentation techniques, and label distribution learning was introduced during model training to address the inevitable label ambiguity between adjacent classes. Five-fold cross-validation was conducted for a comprehensive evaluation of our method. The sensitivity, specificity, and accuracy of the CNN model based on lateral cephalometric radiographs were 83.99, 92.44, and 90.33%, respectively. The accuracy of the model with profile photographs was 83.39%. The accuracy of both CNN models was improved to 91.28 and 83.98%, respectively, while the overfitting decreased after addition of label distribution learning. Previous studies have been based on adult lateral cephalograms. Therefore, our study is novel in using deep learning network architecture with lateral cephalograms and profile photographs obtained from children in order to obtain a high-precision automatic classification of the sagittal skeletal pattern in children.

## 1. Introduction

Orthopedic treatment can guide the normal craniofacial growth of patients who manifest skeletal malocclusions during the growing stage [1]. Therefore, an accurate diagnosis of skeletal malocclusion in children is of great clinical significance. In fact, sagittal skeletal classification is the most important factor for orthodontists to consider when diagnosing skeletal discrepancies and creating a treatment plan for patients [2].

In clinical practice, orthodontists often use lateral cephalograms and profile photographs in order to evaluate the sagittal skeletal pattern of a particular individual. The sagittal skeletal pattern can be classified into classes I, II, and III, according to the ANB angles of the A, N, and B points [3,4]. Much of the literature has led to the supposition that the soft-tissue facial form may be an accurate representation of the underlying skeletal pattern to explore the possibility of ruling out the need for additional radiation exposure. However, the concept of whether the sagittal skeletal pattern can be accurately assessed by a soft tissue profile is controversial [5,6,7,8].

Traditionally, the diagnosis of sagittal skeletal patterns relies on manual detection measurements. However, cephalometric analysis is a time-consuming and laborious process that requires professional training and repeated practice [9]. In addition, errors and bias are inevitable due to the subjective nature of defining the landmarks manually. Therefore, at present, the rapid early diagnosis and treatment of malocclusions in children has become a research hotspot.

In recent years, artificial intelligence (AI) technology based on convolutional neural network (CNN) has been widely applied in various fields [10,11]. Moreover, CNN-based artificial intelligence technology has recently been acknowledged as an effective and trustworthy tool for the diagnosis of medical images [12,13,14]. In orthodontics, several studies have investigated automated lateral cephalometric radiograph landmark identification [15,16,17,18,19]. Arik et al. [15] used a CNN model to automatically detect landmarks on lateral cephalograms, while Yoon et al. used a cascaded CNN for landmark detection for the cephalometric analysis [16]. The application of deep learning algorithms to cephalometric analysis has shown better performance, although many of these studies have focused on detecting landmarks, which then need to be measured and diagnosed in a similar way to conventional cephalometric approaches.

However, many areas of medical research, including breast cancer, lung disease, sagittal ossification of the skull (CSO), and the use of bone segmentation in CT scans [20,21,22,23,24], have successfully used a direct diagnostic system based on convolutional neural networks that uses only X-ray images without providing additional information. Considering the high potential for errors and bias associated with these conventional diagnostic methods, Yu et al. used a landmark detection to create an automatic, one-step, end-to-end deep learning system for skeleton classification in 2020. His method had an accuracy of over 90% [25]. Furthermore, the model performance of four distinct CNN algorithms for automatic sagittal classification on lateral cephalograms was compared [26]. Other researchers subsequently demonstrated that for classification of the sagittal skeleton, the Deep CNN-based AI model performed better than the automatic tracking AI program [27].

From the related work on classification of sagittal skeletal patterns, it can be seen that the existing automatic classification methods were based on the lateral cephalogram data of adult patients. Due to the difficulties associated with sample collection [25], there has not been an automatic sagittal skeletal classification model specifically for child patients, nor was there a classification model of sagittal skeletal patterns based solely on profile photographs.

In order to realize early diagnosis of malocclusions in children, a reduction of unnecessary X-ray radiation, relief of medical resources, and a reduction in the subjective diagnosis of skeletal deformity, in this study, we proposed the establishment of a deep learning method. This was based on using two different datasets in order to automatically classify and diagnose the sagittal bone surfaces in children. Firstly, 4 SOTA models were pre-trained on ImageNet-1k, and these were used to perform fine-tuning of the constructed dataset, and then the optimal performance model was selected for subsequent studies. Although migration learning can partially alleviate the problems associated with data scarcity, some data enhancement was still necessary. To counteract labeling ambiguity, the concept of label distribution learning was included in the model during the training process [28,29]. The label distribution lessens the impact of uncertainties as well as incorrect labeling while also naturally describing the link between all the variables.

Our experiments showed that our deep learning-based model achieved a classification accuracy of more than 90% on the lateral cephalograms. However, due to the compensation needed for soft tissue profiling of bone deformities, the classification accuracy of the profile photographs was approximately 80%. However, the diagnostic accuracy of skeletal Class III malocclusions was greater than 90%.

## 2. Materials and Methods

**Figure 1:**
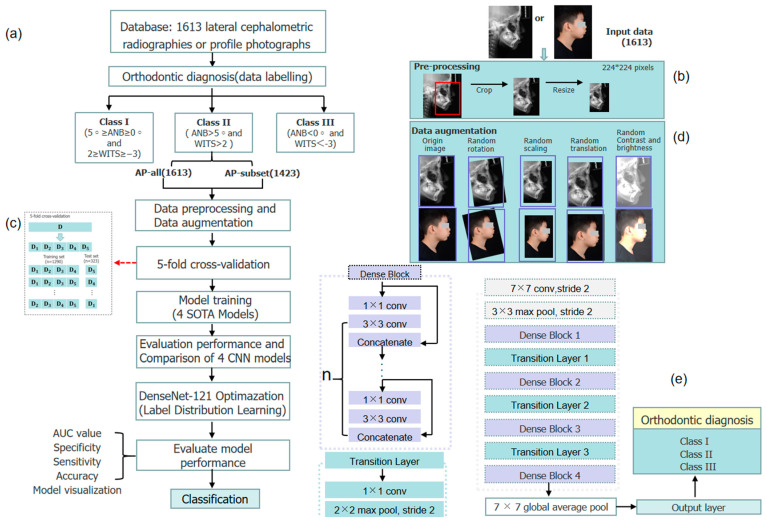
An overview of the model building process and the deep learning model architecture. (**a**) A flow chart of the deep learning model building process. (**b**) A schematic diagram of data preprocessing. (**c**) A schematic diagram of 5-fold cross-validation. (**d**) A schematic diagram of data augmentation. (**e**) A diagnostic model architecture based on the DenseNet-121 model.

This study was exempt from IRB approval, and it was reviewed and confirmed by the Ethics Committee of Guangxi Medical University College of Stomatology (No. 2022091). All the procedures were carried out in conformity with the rules and regulations that applied. The complete methodology used is presented as a schematic diagram in Figure 1a.

### 2.1. Data Collection

A total of 1613 pediatric patients’ datasets (mean age 11.28 ± 1.97 years, range 4–14 years, 797 males, 816 females) were obtained from the Orthodontics Department of Guangxi Medical University College of Stomatology between January 2019 and December 2021 for the present study. Lateral cephalograms and 90° profile photographs, which were taken before orthodontic treatment, were used. Non-standardized and low-resolution images were excluded. The lateral cephalograms were captured with a Myriad Hyperion X9 (Safelite Group, Cormano, Italy), with original images of either 2460 × 1950 or 1752 × 2108 pixels at 0.1 mm/pixel resolution. The 90° profile photographs were captured with a Nikon D7200, with original images of 2510 × 2000 pixels at 0.1 mm/pixel resolution. All images used were in JPG format.

### 2.2. Data Annotation

#### 2.2.1. Diagnostic Criteria

Point A–nasion–point B (ANB) and WITS appraisal are 2 common methods for diagnosing the sagittal skeletal relationship. ANB refers to the anterior-posterior (AP) relationship between the maxilla and mandible, as measured by the angle formed by point A, the nasal root point, and point B [3,4]. The WITS assessment is an analytical method used to categorize sagittal skeletal relationships that indicate the severity of anterior-posterior jaw dissonance. For this technique, the maximal cusp interposition must be used to vertically project points A and B into the occlusal plane [30]. WITS describes a basal connection independent of the anterior cranial base angle, which distinguishes it from ANB measurement [31]. According to the typical mean values of ANB and WITS in the Chinese population, all radiographs in this study were divided into three categories [32,33], i.e., skeletal class I (5° ≥ ANB ≥ 0° and 2 ≥ WITS ≥ −3), skeletal class II (ANB > 5° and WITS > 2), and skeletal class III (ANB < 0° and WITS < −3). Following manual measurements, the classification of which was carried out independently by three orthodontists with a total of 20 years of working experience. If two experts had different judgments on a particular image, then the image would be re-assessed by all experts after a discussion. Classification and labeling of profile photographs were performed in accordance with the corresponding radiographs.

#### 2.2.2. Data Preprocessing and Data Augmentation

To lessen interference from other anatomical structures, the image regions involving points A, N, and B (1500 × 800 pixels) were automatically cropped from the original image by using YOLO v 5. The cropped images were then scaled down to 224 × 224 pixels in size. Label ambiguity was inevitable for samples close to the boundary of two stages, even with meticulous annotation. Therefore, the following data augmentation techniques [34] were also randomly used in this study in order to prevent overfitting of the model on a small dataset: Random rotation, random scaling, random translation, and random changes of contrast and brightness. Figure 1d depicts a schematic diagram of the data augmentation procedure. In each training epoch, the training set data had a 50% probability of data augmentation, generating a maximum of 85,425 (1139 × 150 × 0.5) new data after 150 training epochs. From the entire dataset (AP-all), we extracted a subset named the AP-subset, which only contained 1423 samples of clear sagittal classification (Table 1). The details of all the datasets used are shown in Table 1.

## 3. CNN Model Training Details and Comparisons

Four representative SOTA models were selected as candidate models for our study: ConvNeXt-T, DenseNet-121, Swin-T, and ResNet-101 [35]. We retrieved the pre-trained models of the four candidate CNN models from the PyTorch torchvision model zoo (https://pytorch.org/vision/stable/, accessed on 27 October 2022). When compressing medical data, the four SOTA models typically employed a transfer learning strategy [36], which leveraged the model parameters that have already been trained on non-medical data. The initial weighting of the model were pre-trained weight parameters matching those on the large-scale ImageNet dataset. That is, we used the model structure and pre-training weights in the torchvision models sub-package to modify the classification layer to fit the number of classes of the datasets we were building.

All layers of the CNN models were trained and upgraded using fine-tuning approaches [37]. Each CNN model in this study was trained with 200/150 epochs using the stochastic gradient descent (SGD) optimizer and cross-entropy loss-of-function after the pre-trained model’s parameters were initialized. Next, the hyper-parameters of the neural network were adjusted several times according to their model’s performance on the validation set. Finally, a personalized set of hyper-parameters, including the learning rate, batch size, momentum, and weight decay, were determined to maximize the capability of the particular CNN. All training procedures in this study were performed on a computer equipped with an NVIDIA GeForce RTX 3080 GPU.

The four CNN models were evaluated by 5-fold cross-validation, in which the AP-all dataset was randomly divided into 5 groups. According to the proportion of label categories, each experimental validation and training set contained 323 and 1290 samples, respectively. Fold 1 was used as the validation set, and the rest were the training sets. The next set of experiments used Fold 2 as the validation set and the rest as the training sets, and thus the five cross-validations were performed in this order.

To evaluate the performance of the four SOTA models, the following indicators were calculated, and these included Params, Floating Point operations (FLOPs), Sensitivity (SN), Specificity (SP), Classification Accuracy (ACC), and the AUC values [38,39,40,41]. One of the most prevalent evaluation measures for classification was accuracy, which was calculated by dividing the number of correctly classified samples by the number of images used. The means and standard deviations of the evaluation metrics are reported. The optimal SOTA model was selected for our subsequent studies by employing lateral cephalograms and profile photographs of patients.

## 4. DenseNet-121 Model Modification

As shown in Table 1, there are actual borderline cases between the three groups, as opposed to decision boundaries produced by the model classification. Currently, there are no widely acknowledged clinical criteria for the classification of borderline cases. Therefore, it is acceptable to determine whether these belong to any classification for a borderline case in skeletal Classes I and II. Hence, label confusion is an inevitable occurrence with a borderline case. Instead of using one-hot labels for borderline circumstances, we applied a label distribution learning technique to address this issue.

### Label Distribution Learning

Instead of using one-hot labels for borderline circumstances, we applied label distribution learning to address this issue. First, we created the set $l=\left\{1,2,3\right\}$, which represented the three classification labels for the skeletal classification. Given an input image, x, the one-hot label of x was defined as $y\left(y\in l\right)$, and the label distribution then converts y to $p=\left\{p_{1},p_{2},p_{3}\right\}$ by a function, where $p_{i}$ represents the probability value of x belonging to $l_{i}$. Herein, we used a Gaussian to transform y:

$$p_{i}=\left\{\begin{array}{c} \frac{1}{2\pi \sigma }exp\left(-\frac{{\left(l_{i}-y+d_{1}\right)}^{2}}{2\sigma^{2}}\right),\;\;y*i=6\\ \frac{1}{2\pi \sigma }exp\left(-\frac{{\left(l_{i}-y+d_{2}\right)}^{2}}{2\sigma^{2}}\right),\;\;others \end{array}\right.$$

**Figure 2:**
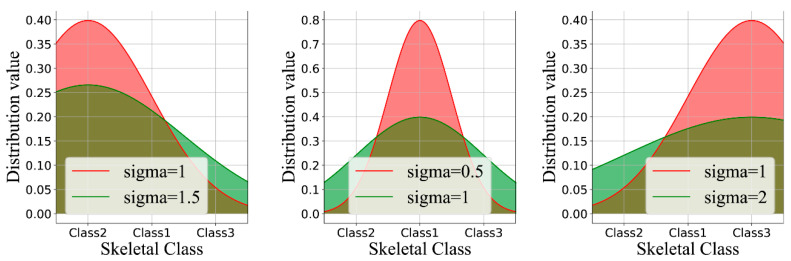
Label distribution learning by varying the labels *y* and *σ*. For better visual effects, the discrete probability values were converted into continuous curves.

σ, $d_{1}$, and $d_{2}$ are the hyper-parameters that need to be set. σ determines the width of the Gaussian function curve, $d_{1}$ and $d_{2}$ represent the distance of different classes, y represents the true label value of the input image x, and $l_{i}$ represents the true label value of the i category. Given that the skeletal Class I in this instance serves as a transitional stage between the skeletal classification II and III and that these two classes are separated by a greater distance, the constraint is made as stated above (Figure 2).

## 5. DenseNet-121 Modified Model Assessment

The performance of this method was then assessed using the metrics listed below: Sensitivity (SN), Specificity (SP), Classification Accuracy (ACC), and Receiver Operating Characteristic (ROC) curve.

Due to label ambiguity, some researchers have argued that adopting an accuracy with one-stage deviation is reasonable. In the present task, considering that label confusion mainly exists between borderline cases, we defined one-stage deviation as follows: It is acceptable to misclassify borderline cases into their adjacent categories. In this study, we reported both normal accuracy and accuracy with a one-stage deviation [39]. The model was also evaluated using 5-fold cross-validation, where the dataset used was randomly divided into 5 parts based on the proportion of label categories. The means and standard deviations of the evaluation metrics are reported.

We created a class activation map (CAM) to better comprehend the learning styles of the model [42]. The CAM visually highlighted the regions of the lateral cephalograms and profile photographs that were most informative in terms of distinguishing between skeletal classifications.

## 6. Results

### 6.1. Sample Statistics

The details of the datasets are given in Table 1. There was no significant difference in age between skeletal Classes I, II, and III (Table S1). For skeletal Classes I, II and III, the mean ANB angles were 2.48°, 5.58°, and −1.85°, while the mean WITS values were −1.27, 2.61, and −6.70, respectively (Table S1), and the observed differences were statistically significant (*p* < 0.001). We discovered 190 cases that did not fit the classification criteria used, and these were borderline cases between the two skeletal classes. The similarities among the cases were particularly challenging for the orthodontists to precisely diagnose the classification label to use (Table S2).

### 6.2. Model Classification Performance

The screening performances of the four models tested in this study are shown in Table 2. Compared with ConvNeXt-T, ResNet-101, and Swin-T, the DenseNet-121 model performed best, with a maximum AUC of 96.80 ± 0.40, although the accuracy of ConvNeXt-T’s was 1% higher than DenseNet-121. However, Params (M) of ConvNeXt-T were more than three times larger than DenseNet-121. In comparison with the model performance of Convnext-T, DenseNet-121 had smaller parameters, the highest AUC value, and an ideal accuracy, so DenseNet-121 was chosen for further experiments.

The sensitivity, specificity, and accuracy of the CNN model for sagittal skeletal classification based on the lateral cephalometric radiographs of our study patients were 83.99, 92.44, and 90.33%, respectively (Table 3). After the borderline cases were eliminated, the ACC of the CNN model increased from 90.33 to 94.05%, while accuracy with a one-stage deviation improved only by 2.05% (Table 3), indicating that a large proportion of misclassified samples in the AP-all dataset were borderline cases.

**Figure 3:**
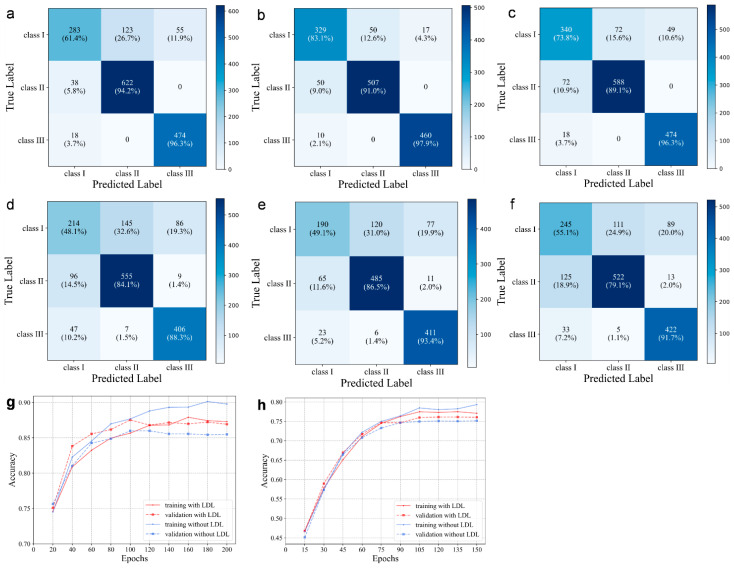
Confusion matrix on AP-all, AP-sub, and AP-all with LDL of lateral cephalograms (**a**–**c**) and profile photographs (**d**–**f**). The training and validation accuracies on AP-all with and without LDL for the lateral cephalograms (**g**) and profile photographs (**h**), respectively.

In order to reduce the impact of borderline cases on the overall accuracy, we introduced the technique of label distribution learning. Figure 3 displays the outcomes of the model-based ablation studies. When the drop rate was 0.2, there was a significant overfitting issue. When a label smoothing value of 0.2 was added, the overfitting decreased, but there was a concomitant decrease in the accuracy of the validation set. After adding label distribution learning, the accuracy of the CNN model increased by 1.0%, and the overfitting was also reduced.

The diagnostic model based on profile photographs also demonstrated close to 80% average clinical performance (Figure 3). The AP-sub of sagittal classification also showed a higher performance with mean values of SN = 76.31 ± 2.02, SP = 88.89 ± 0.85, and ACC = 85.49 ± 1.12 (Table 2). Similarly, the overall accuracy was improved by 0.6% after label distribution learning for the borderline cases, and the overfitting decreased.

**Figure 4:**
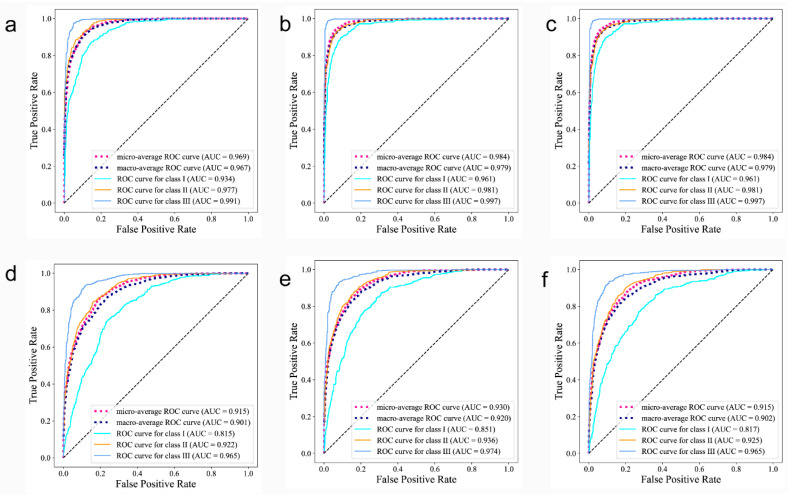
Receiver operating characteristic curves on AP-all, AP-sub and AP-all with LDL of lateral cephalograms (**a**–**c**) and profile photographs (**d**–**f**). The area under the curves (AUCs), which is a measure of the general performance, are shown in parentheses.

Figure 4 shows the ROC (receiver operating characteristic) curves, which integrated the sensitivity and specificity of the classification model, and these can accurately describe the model performance even when the ratios of positive to negative samples were not proportional. An AUC (area under curve) refers to the area under the ROC curve, which quantifies the model’s performance based upon the ROC curve. With most AUC values far exceeding 0.9, from a clinical epidemiologic perspective, we concluded that the proposed medical diagnostic system is able to accurately diagnose a patient’s skeletal class.

**Figure 5:**
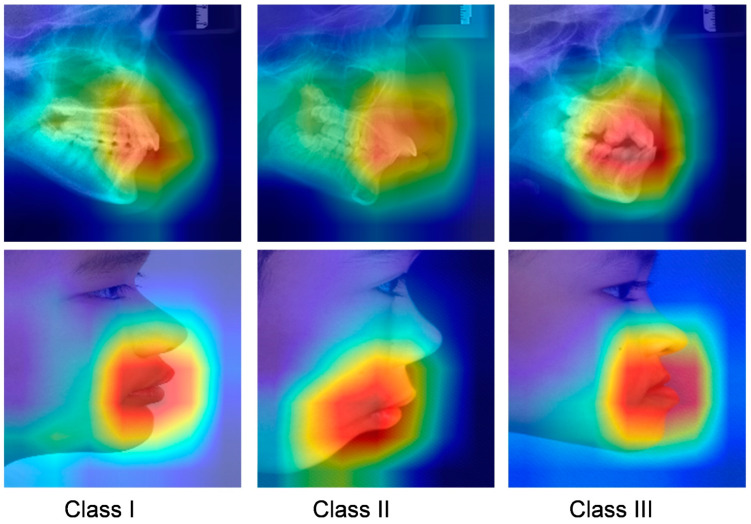
Grad-CAM visualization of images in different skeletal classifications. Red represents high attention, and blue means low attention.

The class activation map is shown in Figure 5. Based on direct visual analysis, we suggest that the main activation regions of the two CNNs were located in the anterior jaw and lip areas, indicating that there is no overfitting in the trained CNNs. However, the activation areas of the three sagittal skeletal patterns were slightly different. The AUC values for each subgroup suggest that all the CNNs showed the highest accuracy in identifying the skeletal Class III pattern, followed by the skeletal Class II and I patterns, as reflected in their confusion matrices and ROC curves (Figure 3 and Figure 4).

## 7. Discussion

In recent years, early orthodontic treatment has emerged as a popular topic, as demand for orthodontic treatment in children has risen. The diagnosis of sagittal skeletal patterns in children is of crucial importance in order to pursue optimal therapeutic strategies. In this study, we trained 2 CNN models for the classification of the sagittal skeletons in children. The models could automatically classify images based only on the lateral cephalograms and profile photographs of patients. This does not only help to guide the junior orthodontists to make a more accurate assessment of the sagittal skeletal pattern of a child but also relieves the more experienced orthodontist from this laborious classification task. In addition, this protocol can enable the parents to make a preliminary assessment of whether their child has a deformity, and they can plan for the possibility of correction therapy.

**Figure 6:**
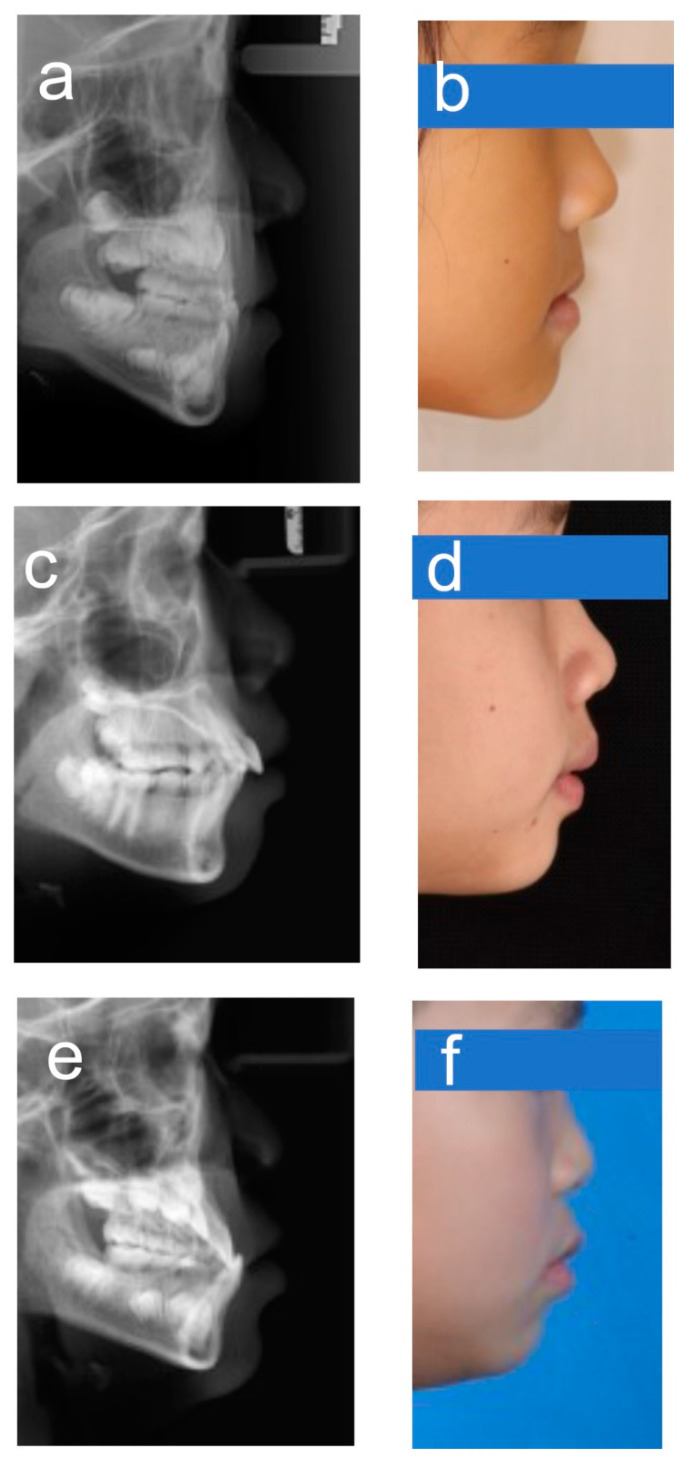
Example images for borderline cases. (**a**,**b**) Class I–III; (**c**,**d**) Class I–II; (**e**,**f**) Class III–I.

Automated diagnosis based on deep learning has gained widespread attention as a practical clinical aid for diagnosis of patients with many different diseases. Previous studies have not reported any automatic skeletal classifications for children whose facial morphology and trends differ significantly from adults. For the automatic categorization application of the sagittal skeletal relationships in children, we trained a representative CNN model, DenseNet-121, by using 1613 lateral cephalograms. Under the conditions used for transfer learning, it was found that this CNN model could be trained without difficulty in under 90 min. It was clear that this CNN model could accurately classify sagittal skeletons of children, with >90% accuracy and >0.96 AUC after the data were subjected to a 5-fold cross-validation test. The accuracy obtained was lower than that observed for adults [25,26], which may be because pediatric patients are likely to be undergoing rapid growth changes. In addition, the shape of the skeletons dramatically varies with age, which can further complicate the diagnosis of younger patients. For instance, individuals with anterior crossbites who are too young to be properly classified may not have visible skeletal deformities yet. Furthermore, despite careful annotation, there would still be many labeling ambiguities in the close proximity of the two classification boundaries, and this can compromise overall accuracy. According to our results, the majority of the misclassified instances were boundary cases that even well-experienced experts had trouble correctly classifying. These cases fell into one of two categories and were mainly found in patients with skeletal Classes II and III (Figure 6). Additionally, we produced a subset of data (AP-sub) that was free of borderline cases and found that the accuracy increased from 90.3 to 94.05%, thus providing additional evidence that the borderline cases affected the overall accuracy of our results.

We introduced a technique of label distribution learning in order to address the problem associated with borderline cases because, in clinical practice, these could not be avoided. By comparing the different graphs obtained in Figure 2, it was evident that in the absence of label distribution learning, the values of the matrix began to spread further from the diagonal ideal situation. The images of stage I can be divided into either stage I or II. Table 2 shows the effects of label distribution learning on the model’s ultimate performance. The accuracy rate rose from 90.33 to 91.28% in the presence of LDL. Label distribution learning naturally describes the uncertainty between genuine images by giving higher confidence to labels obtained, and these were closer to the data. Since the diagnosis in these circumstances can be made between adjacent stages, we performed a one-stage deviation calculation and obtained an accuracy of up to 92.95%.

Several reports in the literature have supported the view that the soft-tissue facial form may be an accurate representation of the underlying skeletal pattern [5,6]. Therefore, we attempted, for the first time, to train a CNN model based on profile photographs for automatic skeletal classification and found that this model had an accuracy of 83.39%, while the highest accuracy of 90.48% was achieved for patients with skeletal Class III. Although the accuracy was lower than in the CNN model based on lateral cephalograms, this was understandable because the soft tissue covering the skeletal surface had a variable thickness, and this may have partially compensated for the existing skeletal incongruity. This was especially so in some borderline cases where soft tissue compensation was very obvious. However, for severe skeletal deformities, the initial evaluation of the skeletal patterns could only be determined by facial profiles. However, this allowed the parents to make a preliminary assessment as to whether their child had the possibility of a craniofacial deformity.

In a medical diagnostic scenario, interpretability is critical for making trustworthy decisions from a human perspective. The visualization of the CAM shows that the neural network model is able to learn a skeleton assessment strategy that is highly consistent with orthodontic clinical criteria. This indicates that the decisions of our model were well interpreted. In addition, the visualization of prediction findings can guide orthodontists to undertake selected re-evaluations in situations where forecasts are dubious.

The datasets created and/or analyzed for the current study are not publicly accessible due to the possibility of violations of patients’ privacy and the presence of confidential information. However, these data can be obtained from the corresponding authors upon justifiable request, although data usage agreements will be required.

## 8. Conclusions

In this study, we propose a novel method based on deep learning for automatic sagittal skeletal classification in children. Firstly, 4 different SOTA models were trained with lateral cephalogram images, and the best-performing model, Densenet-121, was selected for subsequent studies. Lateral cephalograms and profile photographic data were used as the input for the model, respectively. Data augmentation and transfer learning were used to optimize the network parameters, and label distribution learning was introduced during the model’s training process to solve the inevitable labeling ambiguity between adjacent classifications. The performance of the proposed method was then verified. From the results obtained, we can see that the deep CNN not only successfully learns the discriminative representation of the lateral cephalograms but also reduces the problem of overfitting by using label distribution learning. Therefore, the deep learning model based on lateral cephalograms can be a useful tool to assist orthodontists in the diagnosis of sagittal skeletal patterns in children. In addition, the deep learning model based on profile photographs had outstanding advantages in the diagnosis of skeletal Class III malocclusions.

Although the proposed method has achieved good classification performance, the proposed algorithms used can still be affected by label confusion, and the overall accuracy of the model still needs to be improved. Firstly, the small datasets remain a key issue. Therefore, future work requires building larger datasets and using multicenter data wherever possible. Furthermore, although the CNN structure can effectively extract the local features of an image, it is difficult to efficiently extract its global features. In the future, we will try to use the CNN-Transformer-based model to learn advanced representations in order to achieve better classification. Secondly, this study is the first step in the study of a clinical decision system for early orthodontic treatment. However, it does have the potential to narrow the gap between orthodontic diagnosis and treatment level, realize the hierarchical diagnosis and treatment of patients, as well as promote precision orthodontics in young children.

## Figures and Tables

**Table 1:** The Numbers of Patient Data Distributed in Each Skeletal Class together with the Descriptive statistics of the samples in this study.

	AP-All	AP-Sub	Borderline Cases
Numbers (n)	1613	1423	190
Class I	501	427	74
Class II	601	507	94
Class III	511	479	22
Parameters			
Age (y, mean ± SD)	11.28 ± 1.97	11.31 ± 2.36	10.81 ± 2.61
ANB angle (°)	2.30 ± 3.86	1.93 ± 1.75	2.94 ± 1.93
WITS (mm)	(−1.49) ± 3.02	(−1.39) ± 2.45	(−1.24) ± 4.17

**Table 2:** Params (M), Flops (G), Accuracy, Sensitivity, Specificity, and AUC values obtained for the 4 CNN models with lateral cephalograms.

Model	Params (M)	FLOPS (G)	Accuracy (%)	Sensitivity (%)	Specificity (%)	AUC Value
Swin-T	28.3	4.49	62.30 ± 2.67	58.11 ± 2.74	80.04 ± 1.43	78.81 ± 1.54
ResNet-101	44.5	7.8	85.49 ± 1.36	84.86 ± 1.62	92.96 ± 0.76	95.57 ± 0.51
DenseNet-121	8.0	2.83	85.49 ± 0.89	83.99 ± 0.81	92.44 ± 0.40	96.80 ± 0.39
ConvNeXt-T	28.6	4.46	86.30 ± 1.31	85.86 ± 1.23	93.14 ± 0.64	96.00 ± 0.39

**Table 3:** Performance of skeletal classifications for Classes I, II, and III with (1) lateral cephalograms and (2) profile photographs on DenseNet-121.

(1) Performance of skeletal classifications for Classes I, II, and III with lateral cephalograms on DenseNet-121.							
	Sensitivity (%)	Specificity (%)	Accuracy (%)	Sensitivity with LDL (%)	Accuracy with LDL (%)	Accuracy Without LDL with One-Stage Deviation (%)	Accuracy with LDL with One-Stage Deviation (%)
AP-all							
Class I	61.40 ± 4.52	95.14 ± 1.78	85.49 ± 0.89	73.75 ± 3.23	86.92 ± 0.91	88.10 ± 1.37	89.52 ± 1.32
Class II	94.24 ± 4.05	87.09 ± 1.65	90.02 ± 0.87	89.09 ± 3.41	91.07 ± 1.29	92.06 ± 1.20	93.11 ± 1.75
Class III	96.34 ± 2.92	95.10 ± 1.73	95.47 ± 0.54	96.35 ± 1.75	95.85 ± 0.57	95.85 ± 0.69	96.21 ± 0.36
Mean	83.99 ± 0.81	92.44 ± 0.40	90.33 ± 0.59	86.40 ± 0.92	91.28 ± 0.61	92.00 ± 1.21	92.95 ± 0.61
Overall			85.49 ± 0.89		86.92 ± 0.91	90.51 ± 2.09	91.94 ± 0.94
AP-sub							
Class I	83.08 ± 1.85	94.16 ± 1.02	91.08 ± 0.72				
Class II	91.02 ± 1.61	94.23 ± 0.97	92.97 ± 0.89				
Class III	97.87 ± 0.95	98.22 ± 0.53	98.10 ± 0.57				
Mean	90.66 ± 0.73	95.54 ± 0.36	94.05 ± 0.48				
Overall			91.08 ± 0.72				
**(2) Performance of skeletal classifications for Classes I, II, and III with profile photographs on DenseNet-121.**							
	**Sensitivity (%)**	**Specificity (%)**	**Accuracy (%)**	**Sensitivity** **with LDL (%)**	**Accuracy** **with LDL (%)**	**Accuracy** **Without LDL** **with One-Stage Deviation (%)**	**Accuracy** **with LDL** **with One-Stage Deviation (%)**
AP-all							
Class I	48.09 ± 6.61	87.23 ± 2.22	76.10 ± 1.57	55.06 ± 2.35	77.13 ± 1.19	78.78 ± 1.64	79.81 ± 1.56
Class II	84.09 ± 2.91	83.21 ± 3.93	83.58 ± 1.67	79.09 ± 4.22	83.77 ± 1.31	85.69 ± 1.69	85.87 ± 1.91
Class III	88.26 ± 1.60	91.40 ± 2.45	90.48 ± 1.63	91.74 ± 1.48	91.05 ± 0.53	90.86 ± 1.87	91.43 ± 0.71
Mean	73.48 ± 2.18	87.28 ± 1.05	83.39 ± 1.34	75.29 ± 0.85	83.98 ± 1.29	85.11 ± 0.15	85.70 ± 0.13
Overall			75.08 ± 2.00		75.98 ± 0.85	80.25 ± 2.70	81.15 ± 2.50
AP-sub							
Class I	49.07 ± 7.69	91.21 ± 1.68	79.47 ± 1.45				
Class II	86.46 ± 3.35	84.76 ± 3.09	85.45 ± 1.33				
Class III	93.41 ± 2.43	90.71 ± 3.50	91.57 ± 1.83				
Mean	76.31 ± 2.02	88.89 ± 0.85	85.49 ± 1.12				
Overall			78.24 ± 1.68				

## Data Availability

All the data generated and analyzed during this study are included in this published article (and its supplementary information files).

## Supplementary Materials

The following supporting information can be downloaded at: https://www.mdpi.com/article/10.3390/diagnostics13101719/s1. Refs. [43,44,45] are cited in the supplementary materials.

## References

[1] Zhao Z.H., Zhou Y.H., Bai Y.X. (2020). Orthodontics.

[2] Proffit W.R., Fields H.W., Larson B., Sarver D.M. (2018). Contemporary Orthodontics-E-Book.

[3] Steiner C.C. (1953). Cephalometrics For You And Me. Am. J. Orthod..

[4] Steiner C. (1959). Cephalometrics In Clinical Practice. Angle Orthod..

[5] Barnett D.P. (1975). Variations In The Soft Tissue Profile And Their Relevance To The Clinical Assessment Of Skeletal Pattern. Br. J. Orthod..

[6] Riedel R. (1957). An Analysis Of Dentofacial Relationships. Am. J. Orthod..

[7] Staudt C.B., Kiliaridis S. (2009). A Nonradiographic Approach To Detect Class Iii Skeletal Discrepancies. Am. J. Orthod. Dentofac. Orthop..

[8] Schwabe S.A., Caldwell S. (2022). Can Anteroposterior Skeletal Pattern Be Determined From A Silhouetted Profile Photograph? A Cross-Sectional Study. J. Orthod..

[9] Gravely J.F., Benzies P.M. (1974). The Clinical Significance Of Tracing Error In Cephalometry. Br. J. Orthod..

[10] Yu Y., Liang S., Samali B., Nguyen T.N., Zhai C., Li J., Xie X. (2022). Torsional Capacity Evaluation Of Rc Beams Using An Improved Bird Swarm Algorithm Optimised 2D Convolutional Neural Network. Eng. Struct..

[11] Yu Y., Li J., Li J., Xia Y., Ding Z., Samali B. (2023). Automated Damage Diagnosis Of Concrete Jack Arch Beam Using Optimized Deep Stacked Autoencoders And Multi-Sensor Fusion. Dev. Built Environ..

[12] Hamet P., Tremblay J. (2017). Artificial Intelligence In Medicine. Metabolism.

[13] Schwendicke F., Samek W., Krois J. (2020). Artificial Intelligence In Dentistry: Chances And Challenges. J. Dent. Res..

[14] Schwendicke F., Golla T., Dreher M., Krois J. (2019). Convolutional Neural Networks For Dental Image Diagnostics: A Scoping Review. J. Dent..

[15] Arık S., Ibragimov B., Xing L. (2017). Fully Automated Quantitative Cephalometry Using Convolutional Neural Networks. J. Med. Imaging.

[16] Yoon H.J., Kim D.R., Gwon E., Kim N., Baek S.H., Ahn H.W., Kim K.-A., Kim S.J. (2022). Fully Automated Identification Of Cephalometric Landmarks For Upper Airway Assessment Using Cascaded Convolutional Neural Networks. Eur. J. Orthod..

[17] Lee H., Park M., Kim J. (2017). Cephalometric Landmark Detection In Dental X-Ray Images Using Convolutional Neural Networks. Proceedings of the Medical Imaging 2017: Computer-Aided Diagnosis.

[18] Torosdagli N., Liberton D.K., Verma P., Sincan M., Lee J.S., Bagci U. (2019). Deep Geodesic Learning For Segmentation And Anatomical Landmarking. IEEE Trans. Med. Imaging.

[19] Nishimoto S., Sotsuka Y., Kawai K., Ishise H., Kakibuchi M. (2019). Personal Computer-Based Cephalometric Landmark Detection With Deep Learning, Using Cephalograms On The Internet. J. Craniofac. Surg..

[20] You L., Zhang G., Zhao W., Greives M., David L., Zhou X. (2020). Automated Sagittal Craniosynostosis Classification From Ct Images Using Transfer Learning. Clin. Surg..

[21] Ahmadi A., Kashefi M., Shahrokhi H., Nazari M.A. (2021). Computer Aided Diagnosis System Using Deep Convolutional Neural Networks For Adhd Subtypes. Biomed. Signal Process. Control.

[22] Belal S.L., Sadik M., Kaboteh R., Enqvist O., Ulén J., Poulsen M.H., Simonsen J., Høilund-Carlsen P.F., Edenbrandt L., Trägårdh E. (2019). Deep Learning For Segmentation Of 49 Selected Bones In Ct Scans: First Step In Automated Pet/Ct-Based 3d Quantification Of Skeletal Metastases. Eur. J. Radiol..

[23] Wang X., Peng Y., Lu L., Lu Z., Bagheri M., Summers R.M. Chestx-Ray8: Hospital-Scale Chest X-Ray Database And Benchmarks On Weakly-Supervised Classification And Localization Of Common Thorax Diseases. Proceedings of the IEEE Conference On Computer Vision And Pattern Recognition.

[24] Zhu W., Lou Q., Vang Y.S., Xie X. (2017). Deep Multi-Instance Networks With Sparse Label Assignment For Whole Mammogram Classification. Proceedings of the International Conference On Medical Image Computing And Computer-Assisted Intervention.

[25] Yu H.J., Cho S.R., Kim M.J., Kim W.H., Kim J.W., Choi J. (2020). Automated Skeletal Classification With Lateral Cephalometry Based On Artificial Intelligence. J. Dent. Res..

[26] Li H., Xu Y., Lei Y., Wang Q., Gao X. (2022). Automatic Classification For Sagittal Craniofacial Patterns Based On Different Convolutional Neural Networks. Diagnostics.

[27] Kim H.J., Kim K.D., Kim D.H. (2022). Deep Convolutional Neural Network-Based Skeletal Classification Of Cephalometric Image Compared With Automated-Tracing Software. Sci. Rep..

[28] Gao B.B., Xing C., Xie C.W., Wu J., Geng X. (2017). Deep Label Distribution Learning With Label Ambiguity. IEEE Trans. Image Process..

[29] (2022). IEEE Journal Of Biomedical And Health Informatics. IEEE J. Biomed. Health Inform..

[30] Jacobson A. (1975). The “Wits” Appraisal Of Jaw Disharmony. Am. J. Orthod..

[31] Bishara S.E., Fahl J.A., Peterson L.C. (1983). Longitudinal Changes In The Anb Angle And Wits Appraisal: Clinical Implications. Am. J. Orthod..

[32] So L.L., Davis P.J., King N.M. (1990). “Wits” Appraisal In Southern Chinese Children. Angle Orthod..

[33] Cooke M.S., Wei S.H. (1988). An Improved Method For The Assessment Of The Sagittal Skeletal Pattern And Its Correlation To Previous Methods. Eur. J. Orthod..

[34] Perez L., Wang J. (2017). The Effectiveness Of Data Augmentation In Image Classification Using Deep Learning. arXiv.

[35] He K., Zhang X., Ren S., Sun J. Deep Residual Learning for Image Recognition. Proceedings of the IEEE Conference On Computer Vision And Pattern Recognition.

[36] Huang G., Liu Z., Van Der Maaten L., Weinberger K.Q. Densely Connected Convolutional Networks. Proceedings of the IEEE Conference On Computer Vision And Pattern Recognition (CVPR).

[37] Kensert A., Harrison P.J., Spjuth O. (2019). Transfer Learning With Deep Convolutional Neural Networks For Classifying Cellular Morphological Changes. Slas Discov..

[38] Dzemidzic V., Sokic E., Tiro A., Nakas E. (2015). Computer Based Assessment Of Cervical Vertebral Maturation Stages Using Digital Lateral Cephalograms. Acta Inform. Med..

[39] Baptista R.S., Quaglio C.L., Mourad L.M., Hummel A.D., Caetano C.A.C., Ortolani C.L.F., Pisa I.T. (2012). A Semi-Automated Method For Bone Age Assessment Using Cervical Vertebral Maturation. Angle Orthod..

[40] Amasya H., Yildirim D., Aydogan T., Kemaloglu N., Orhan K. (2020). Cervical Vertebral Maturation Assessment On Lateral Cephalometric Radiographs Using Artificial Intelligence: Comparison Of Machine Learning Classifier Models. Dentomaxillofacial Radiol..

[41] Kim E.G., Oh I.S., So J.E., Kang J., Le V.N.T., Tak M.K., Lee D.W. (2021). Estimating Cervical Vertebral Maturation With A Lateral Cephalogram Using The Convolutional Neural Network. J. Clin. Med..

[42] Zhou B., Khosla A., Lapedriza A., Oliva A., Torralba A. Learning Deep Features For Discriminative Localization. Proceedings of the IEEE Conference On Computer Vision And Pattern Recognition.

[43] Memar P., Faradji F. (2017). A novel multi-class EEG-based sleep stage classification system. IEEE Trans. Neural Syst. Rehabil. Eng..

[44] Yang Y. (1999). An evaluation of statistical approaches to text categorization. Inf. Retr..

[45] Liu Z., Lin Y., Cao Y., Hu H., Wei Y., Zhang Z., Lin S., Guo B. Swin transformer: Hierarchical vision transformer using shifted windows. Proceedings of the IEEE/CVF International Conference on Computer Vision.

