# Supplementary material for: Automated Sagittal Skeletal Classification of Children Based on Deep Learning
Source: Diagnostics (Basel). 2023 May 12;13(10):1719. doi: 10.3390/diagnostics13101719 (PMC10217682; doi:10.3390/diagnostics13101719)
Supplement: Supplementary file 1 [file diagnostics-13-01719-s001.zip › diagnostics-2361182-supplementary.pdf]

Automated Sagittal Skeletal Classification of Children Based on Deep Learning

Lan Nan, Min Tang, Bohui Liang, Shuixue Mo, Na Kang, Shaohua Song, Xuejun Zhang and Xiaojuan Zeng

Appendix

Methods Details

Model Training Details

The structure of model layers and hyperparameters were optimized to efficiently combine and analyze the features from the cephalogram information. Pytorch was used as the framework and was trained using SGD optimizer, with cosine learning rate decay and standard parameters (momentum = 0.9, weight decay = 0.05, learning rate = 0.0001). For label distribution learning, the hyperparameters as follows: $\sigma = 0.7$, $d_1 = 2$, $d_2 = 0.5$.

Statistical Analysis

Confusion matrices, diagnostic accuracy, sensitivity, specificity, the receiver operating

characteristic (ROC) curves, and the area under the curve (AUC) were used to test the system performance. We run each method with 5-fold cross-validation and report the average, standard deviation. The following algorithms were used for the calculation of accuracy, sensitivity, and specificity (Memar and Faradji 2018):

Accuracy (AC) = (TP + TN)/(TP + FN + TN + FP) (%)
Sensitivity (SN) = TP/(TP + FN) (%)
Specificity (SP) = TN/(TN + FP) (%)
WHERE TP denotes true positives, TN denotes true negatives, FP denotes false positives, and FN denotes false negatives.

The ROC curves and the AUCs were calculated for each skeletal class. Two types of averages: micro-average and macro-average were used in this work (Yang 1999). AUC is an effective and comprehensive measure of sensitivity and specificity for assessing the inherent validity of a diagnostic test and the overall performance of the ROC curve. Additionally, high AUC values confirm the accuracy by which the model can distinguish patients with or without diseases from a wide range of operating points.

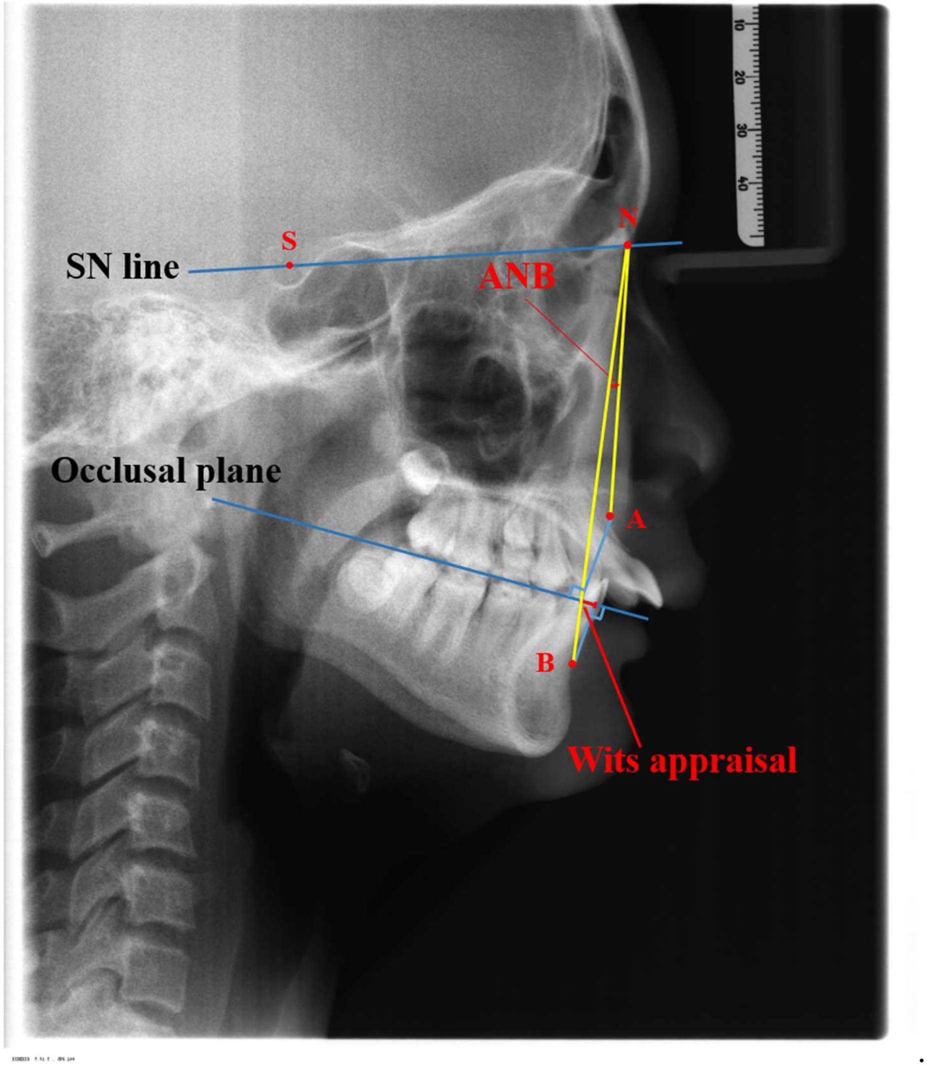

Figure S1. Cephalometric analysis used in this study. Landmarks. S, sella; N, nasion; A, subspinale; B, supramentale. ANB and Wits appraisal are described in the figure.

Table S1. The gender, age, ANB angle and wits value of the three sagittal skeletal classifications.

Parameters	Class I (n = 501)	Class II (n = 612)	Class III (n = 500)	Total (n = 1613)	*p*
Gender (n)					
Male	249	302	246	797	
Female	252	310	254	816	
Age (y, mean ± SD)	11.81 ± 1.76	11.53 ± 1.68	10.58 ± 2.24	11.28 ± 1.97	
ANB angle (°)	2.48 ± 1.55	5.58 ± 1.77	(−1.85) ± 2.89	2.30 ± 3.86	<0.001
WITS (mm)	(−1.27) ± 2.74	2.61 ± 2.79	(−6.70) ± 3.51	(−1.49) ± 5.02	<0.001

Table S2. The gender, age, ANB angle and wits value of the borderline cases.

Parameters	Class I tendency (n = 65)	Class II tendency (n = 103)	Class III tendency (n = 22)	Total (n = 190)
Gender (n)				
Male	31	54	12	97
Female	34	49	10	93
Age (y, mean ± SD)	10.61 ± 2.55	11.31 ± 2.36	8.95 ± 2.98	10.81 ± 2.61
ANB angle (°)	1.76 ± 1.50	4.23 ± 0.75	(−0.08) ± 1.74	2.94 ± 1.93
WITS (mm)	(−3.60) ± 3.63	1.06 ± 2.45	(−5.51) ± 4.97	(−1.24) ± 4.17

Hyper Parameter Setting

Image size	224 × 224
batch size	64
training epochs	200/150
optimizer	SGD
drop rate	0.2/0.0
learning rate schedule	Cosine Annealing
base learning rate	3e-4/1e-4
weight decay	0.05
σ	0.8
d_1	5
d_2	0.5

Average performance comparison in standard metrics of average ACC and average AUC over all-dataset

Model	Image Size	Params (M)	FLOPS (G)	AUC Value	ACC(%)
ResNet-101 [1]	224^2	44.5	7.8	78.81 ± 1.54	62.30 ± 2.67
DenseNet-121	224^2	8.0	2.83	95.57 ± 0.51	85.49 ± 1.36
Swin-T [3]	224^2	28.3	4.49	96.80 ± 0.39	85.49 ± 0.89
ConvNeXt-T	224^2	28.6	4.46	96.00 ± 0.39	86.30 ± 1.31

References

1. Memar, P.; Faradji, F. A novel multi-class EEG-based sleep stage classification system. IEEE Trans. Neural Syst. Rehabil. Eng. 2017, 26, 84–95.
2. Yang, Y. An evaluation of statistical approaches to text categorization. Inf. Retr. 1999, 1, 69–90.
3. He, K.; Zhang, X.; Ren, S.; Sun, J. Deep residual Learning for Image Recognition. In Proceedings of the IEEE Conference on Computer Vision and Pattern Recognition, Las Vegas, NV, USA, 26–30 July 2016; pp. 770-778.
4. Liu, Z.; Lin, Y.; Cao, Y.; Hu, H.; Wei, Y.; Zhang, Z.; Lin, S.; Guo, B. Swin transformer: Hierarchical vision transformer using shifted windows. In Proceedings of the IEEE/CVF International Conference on Computer Vision, Montreal, BC, Canada, 11–17 October 2021; pp. 10012–10022.
